# Selection signatures and landscape genomics analysis to reveal climate adaptation of goat breeds

**DOI:** 10.1186/s12864-024-10334-x

**Published:** 2024-04-29

**Authors:** Weifeng Peng, Yiyuan Zhang, Lei Gao, Wanlu Shi, Zi Liu, Xinyu Guo, Yunxia Zhang, Bing Li, Guoyin Li, Jingya Cao, Mingsheng Yang

**Affiliations:** 1 College of Life Science and Agronomy, Zhoukou Normal University, Zhoukou, China; 2 State Key Laboratory for Sheep Genetic Improvement and Healthy Production, Xinjiang Academy of Agricultural and Reclamation Sciences, Shihezi, China

**Keywords:** Goat, Selection pressure, Environment, Signatures of Selection, SNP

## Abstract

Goats have achieved global prominence as essential livestock since their initial domestication, primarily owing to their remarkable adaptability to diverse environmental and production systems. Differential selection pressures influenced by climate have led to variations in their physical attributes, leaving genetic imprints within the genomes of goat breeds raised in diverse agroecological settings. In light of this, our study pursued a comprehensive analysis, merging environmental data with single nucleotide polymorphism (SNP) variations, to unearth indications of selection shaped by climate-mediated forces in goats. Through the examination of 43,300 SNPs from 51 indigenous goat breeds adapting to different climatic conditions using four analytical methods: latent factor mixed models (LFMM), F-statistics (Fst), Extended haplotype homozygosity across populations (XPEHH), and spatial analysis method (SAM), A total of 74 genes were revealed to display clear signs of selection, which are believed to be influenced by climatic conditions. Among these genes, 32 were consistently identified by at least two of the applied methods, and three genes (*DENND1A*, *PLCB1*, and *ITPR2*) were confirmed by all four approaches. Moreover, our investigation yielded 148 Gene Ontology (GO) terms based on these 74 genes, underlining pivotal biological pathways crucial for environmental adaptation. These pathways encompass functions like vascular smooth muscle contraction, cellular response to heat, GTPase regulator activity, rhythmic processes, and responses to temperature stimuli. Of significance, GO terms about endocrine regulation and energy metabolic responses, key for local adaptation were also uncovered, including biological processes, such as cell differentiation, regulation of peptide hormone secretion, and lipid metabolism. These findings contribute to our knowledge of the genetic structure of climate-triggered adaptation across the goat genome and have practical implications for marker-assisted breeding in goats.

**Supplementary Information:**

The online version contains supplementary material available at 10.1186/s12864-024-10334-x.

## Background

The genetic evolution of species is profoundly shaped by the climate environment, representing a key factor in their sustained genetic adaptation [1]. Species distribution is affected by climate, and any climate alterations will affect the genetic variation of different populations [2]. Climate changes can impact species distribution, subsequently influencing genetic diversity within populations. Investigations across a spectrum of species, such as goats [3], sheep [4], and humans [5], have elucidated that climate fluctuations can give rise to divergences in both phenotype and genotype for populations and individuals. The examination of climate-driven selective pressure is a key focus in evolutionary biology, shedding light on the genetic mechanisms underlying local adaptation and speciation in the face of changing climates. Previous research has revealed instances of adaptation to climate in characteristics such as thermal response [6], body size [7], and pigmentation [8, 9]. In recent years, molecular biology, genetics, and bioinformatics have made great strides, which has strongly advanced animal genomics research. Significant advancements have been achieved in studying the environmental adaptation of domesticated animals such as horses [10], sheep [11], goats [12], and cattle [13]. Key genes associated with economic traits and environmental resilience have been identified in this research. The history of livestock populations is characterized by domestication and selective breeding to enhance desirable production traits. This evolutionary process can be elucidated by comparing genomic patterns of SNP variability, particularly among different breeds. This has allowed for the identification of numerous genomic regions and genes subjected to selection sweeps [14–16]. Many studies have used methods like Fst-based outliers and selective sweep tests to examine allele frequency variations [17, 18]. However, these investigations have not typically integrated genomic and environmental data. As a result, it remains challenging to link selection signatures to specific spatially varying selective pressures, such as particular environmental variables. In recent years, landscape genomics has introduced several approaches to detect adaptations to climatic conditions by examining the relationship between SNP alleles and climate variables, such as the spatial analysis method (SAM) [19, 20] and latent factor mixed models (LFMM) [21, 22]. These approaches have their strengths and limitations, rooted in their underlying assumptions. Through the application of these methods, several studies have effectively revealed genetic adaptation to various climatic pressures by investigating genome-environment correlations in different organisms [4, 6].

Based on archaeological and genetic evidence, the initiation of goat domestication can be dated back to around 11,000 years ago in the Fertile Crescent region, with its roots connected to the distinct wild ancestor, the Bezoar (*Capra aegagrus*) [23]. Goats accompanied human migrations, spreading from Asia to Europe, Africa, the Americas, and Oceania [24]. Over millennia of migration and evolution, domestic goats have adapted to various environments, from the frigid regions of northern Europe to the hot climates of Africa, the arid deserts of North Africa to the humid areas of Southeast Asia, and from low-altitude plains to high-altitude plateaus [25–28]. These adaptations underline their remarkable ability to thrive in diverse climatic and environmental conditions. Goats have played an essential role in human society, offering valuable resources like cashmere, milk, and meat, contributing to agriculture, economy, and culture. Unfortunately, extreme temperatures and humidity can reduce livestock productivity, increasing mortality rates and causing economic losses to the livestock industry [29]. Therefore, extensive research on the climate adaptability of goats can provide theoretical insights to protect and leverage their economically valuable traits, disease resistance, and other remarkable attributes, thus advancing the goat industry.

In this study, distinct strategies were employed to pinpoint regions undergoing artificial and environmental selection of goat breeds using the AdaptMap goat dataset, which comprises over 3,000 animals collected globally and genotyped with the Caprine SNP50 BeadChip [24]. 51 native goat breeds were selected for selection tests, which were conducted using four methods, each operating on different assumptions. The study utilized genetic differentiation analysis of single nucleotide polymorphisms (SNPs) and investigated genetic-environmental correlations by analyzing genomic data alone or in conjunction with environmental data. Our study aims to analyze the genetic legacy passed down through centuries of climate-induced adaptations by identifying selection signatures on a genome-wide scale. These discoveries will contribute to our comprehension of the genetic foundation of adaptive evolution in reaction to climate changes, thus supporting functional genomics, selective breeding methodologies, and the formulation of conservation strategies to address rapid global climate changes in goats and other livestock.

## Methods

### Goat breeds and samples

**Fig. 1:**
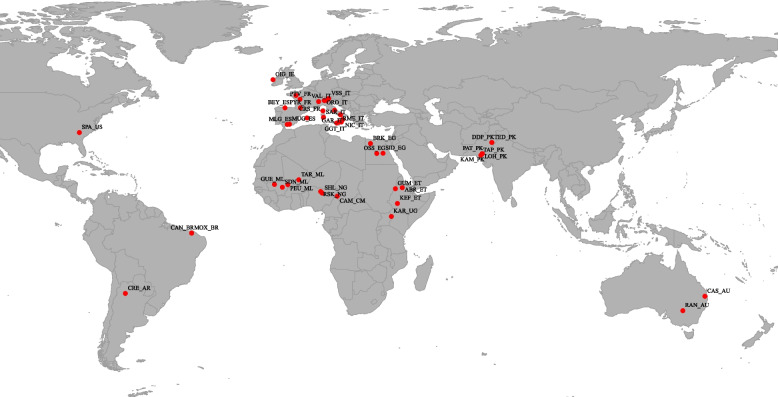
Geographical distribution map of 51 goat breeds in this study

From the 130 domestic goat breeds included in the AdaptMap goat dataset [24], 51 native breeds were specifically chosen (Fig. 1 and Table S1). These selected breeds underwent sampling and genotyping through the Illumina GoatSNP50 BeadChip, which encompasses 53,347 SNPs. The selection process was guided by two primary considerations: the breeds belong to old, autochthonous breeds, and their alignment with environmental and genetic clustering. For each breed, kinship coefficients between individuals in pairs were calculated [30]. To reduce the influence of variations in sample sizes on the resulting estimates, the selection process involved choosing the 20 individuals with the lowest pairwise relatedness from breeds containing more than 20 samples [4]. Details of the number of samples, sampling locations, and coordinates are included in Table S1. The plink software [31] was utilized to perform animal and SNP quality filtering based on following the criteria [24]: (1) individuals with a call rate < 96%; (2) SNP with a < 95% genotyping rate; (3) SNP with a minor allele frequency (MAF) > 0.05; (4) SNP with physical locations on autosomes. After eliminating SNP and individuals that did not meet the criteria, our dataset was comprised of 43,300 SNP (before quality control 53,347 SNPs) and 1,020 individuals from 51 breeds.

### PCA based on environmental variables

**Fig. 2:**
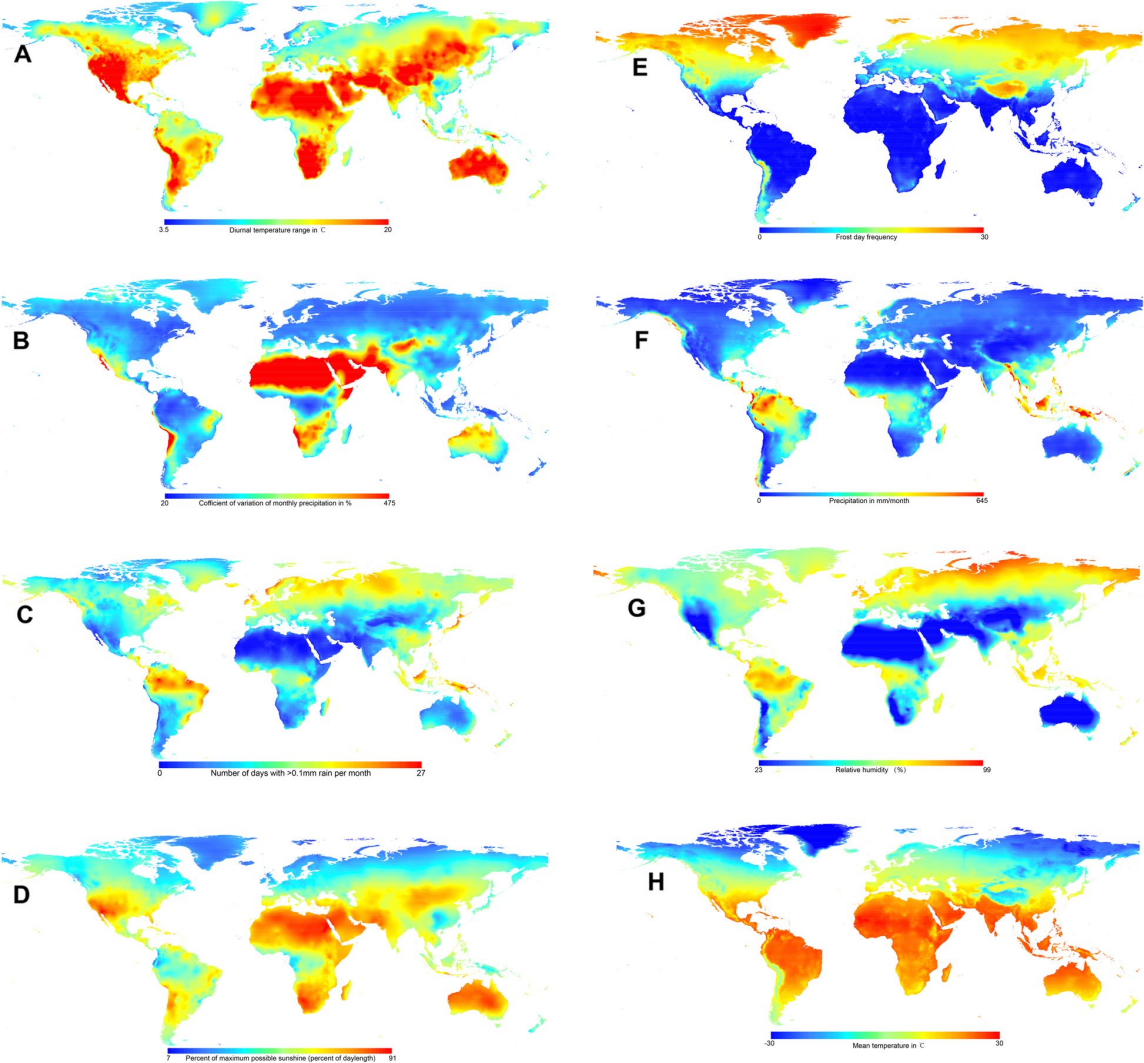
Climatic data used in this study. Maps display the geographical distribution of annual mean values for the eight climate variables. **A** Diurnal temperature range (DTR); **B** Coefficient of variation of monthly precipitation (PRCV); **C** Number of days with > 0.1 mm rain per month (RDO); **D** Percent maximum possible sunshine (SUN); **E** Frost day of frequency (FRS); **F** Precipitation in mm/month (PR); **G** Relative humidity (REH); **H** Mean temperature (TMP)

**Fig. 3:**
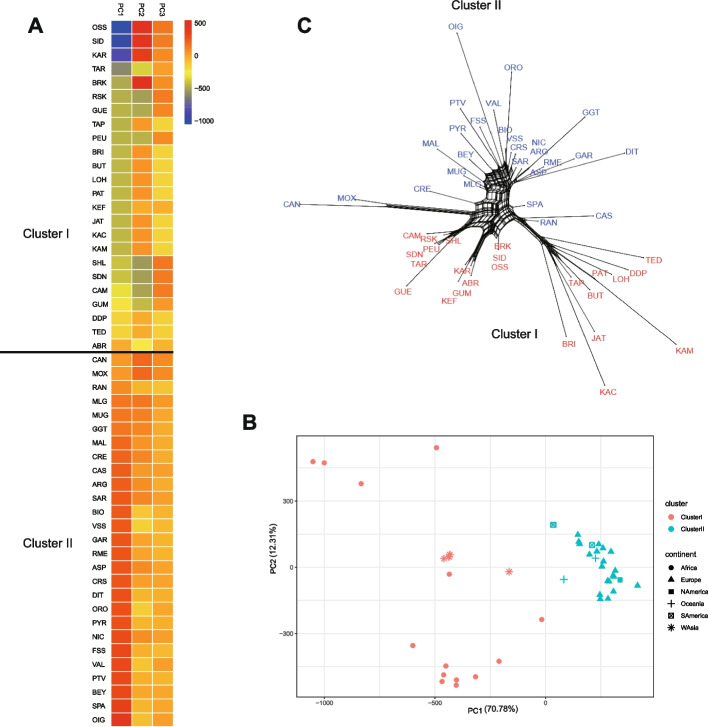
PCA of environmental variables and Genetic relationship of 51 native goat breeds. **A** Heat strips for each of the first three PCs are shown for the 51 goat breeds assigned to the two clusters (I and II). **B** the score plots of PC1 versus PC2 for the 51 native goat breeds: breeds from Africa and West Asia clustered together in Cluster I, and breeds from Europe, America, and Oceania formed Cluster II. **C** Genetic relationship between the 51 native goat breeds based on Reynolds’ genetic distance. Two clusters I and II are indicated in red and blue, respectively

Data on environmental variables from the Climatic Research Unit of Norwich (CRU CLv.2.0), covering the period 1961 to 2001, was downloaded from the climate data set (https://crudata.uea.ac.uk/cru/data/hrg/, last accessed June 3, 2023) [32]. Climate data was composed of latitude/longitude grids with a resolution of 10 min, containing yearly mean and monthly values of eight variables across global land areas. Monthly parameters of the variables were included in this study to account for seasonal fluctuations, such as vegetation growth and lambing. The climate variables applied in this study were similar to the study by Lv et al. 2014, encompassing temperature, ground frost, precipitation, relative humidity, and sunshine [4] (Fig. 2 and Table S1). These climate data were acquired by utilizing a *raster* function in the R package based on the longitude and latitude coordinate data [26]. In addition, PCA was calculated to differentiate breeds based on a total of 104 environmental parameters using the princomp function of R software (Fig. 3A and B).

### Population structure analysis

**Fig. 4:**
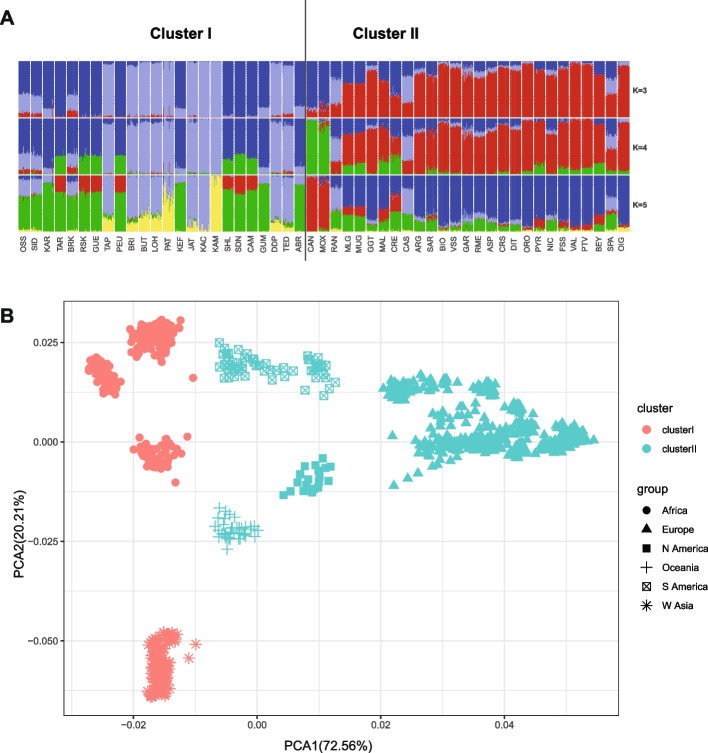
Population genetic structure among the 51 goat breeds with worldwide origins. **A** Bayesian analysis base on K = 3–5; Each animal is depicted by a solitary vertical line segmented into K colors, with K representing the assumed number of clusters. The colored section indicates the estimated proportion of the individual's membership in that cluster, averaged across 10 runs for each K value ranging from 3 to 5. **B** Principal component analysis (PCA) using SmartPCA from the EIGENSOFT v5.0 package

To address biases that may result from population structure, the genetic relationships among 51 goat breeds were examined to remove closely related breeds from the analysis. The Arlequin v3.11 software package [33] was employed to compute the pairwise Reynolds’ genetic distances between populations. The Reynolds' genetic distance pairwise matrix was calculated using 22,861 SNPs, applying the LD pruning algorithm in the plink indep-pairwise command (parameters: 50 5 0.05). This method entails assessing LD between SNPs in windows of 50 markers and removing one SNP from each pair when the *r2* LD index exceeds 0.05. Subsequently, a genetic relationship network was established among breeds through a neighbor-net analysis conducted with the SplitsTree package v4.12 [34] (Fig. 3C). Utilizing the program STRUCTURE v2.3.4, a Bayesian clustering method was employed to evaluate genetic structure among populations [35]. This method utilizes multilocus genotypes for inferring population genetic structuring and determining the number of genetic clusters (K). 10 runs for each K value ranging from 3 to 5 were conducted, employing a model of admixture and correlated allele frequencies in the program (Fig. 4A). Moreover, principal component analysis on the individuals based on SNP data was performed using the SmartPCA program (http://www.hsph.harvard.edu/alkes-price/software/) from the EIGENSOFT package (Fig. 4B).

### Screening for SNPs and genomic regions under selection

**Fig. 5:**
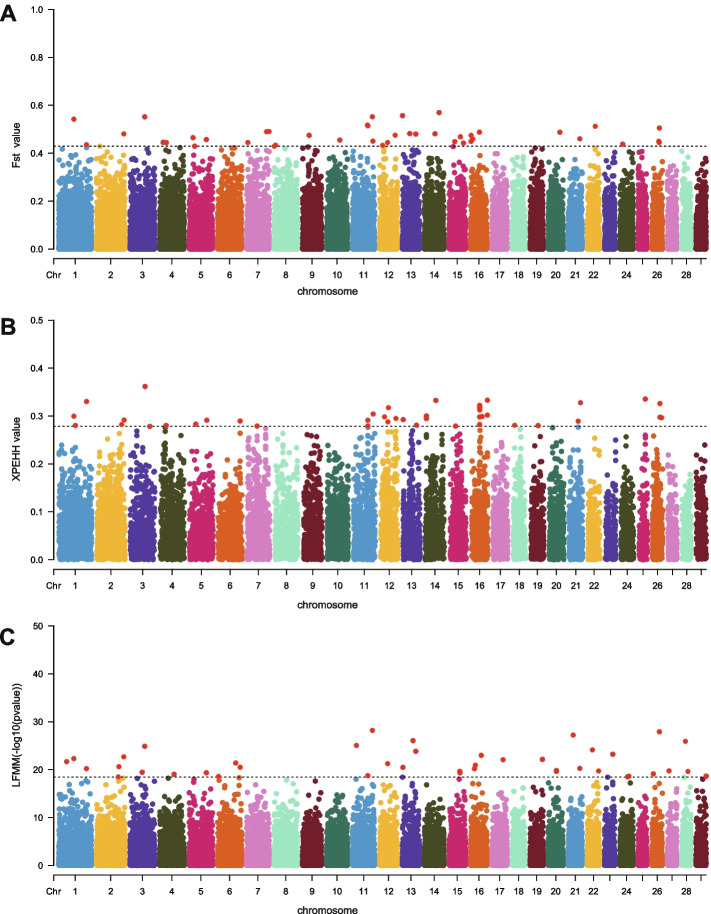
Genome-wide selection signatures identified through Fst, XPEHH, and LFMM tests. **A** The genetic differentiation between two clusters (I vs II) among 51 breeds evaluated by analyzing the genome-wide distribution of Fst values. Red color represent the top 1% candidate SNPs. **B** Genome-wide distribution of selection signatures detected by XPEHH using two clusters (I vs II). The red SNPs represents the threshold levels of top 1%. **C** The distribution of significance values (-log10(P)) examined for correlations between SNP frequencies and environmental variables in the LFMM test.; The red SNPs represents the threshold levels of top 1%

To pinpoint genomic regions that may be under selection, Fst values were calculated using 43,300 SNPs. This analysis was applied to two clusters, cluster I and cluster II, which were distinctively determined by the PCA of environmental data, Reynold’s genetic distance and population structure analysis (Figs. 3 and 4). The Fst single locus analysis technique were utilized, as suggested by Weir and Cockerham (1984) to evaluate the degree of population differentiation [36]. Fst values was calculated with VCFtools, utilizing a non-window approach [37]. Significant markers were empirically determined to be the top 1% of SNPs in this study (Fig. 5A). In contrast to the Fst method, XPEHH operates as a haplotype-based approach. SHAPEIT was utilized to construct haplotypes in each breed initially [38], and subsequently, SELSCAN was used to compute XPEHH statistics for each population pair [39]. After normalizing the XPEHH values, which approximately followed a normal distribution. A significance test of the standard normal distribution (*p* < 0.05) was utilized to assess the differences in variations attributed to selection among populations. Selection is denoted by positive XP-EHH values in the observed population and negative values in the reference population. Additionally, the top 0.1% XPEHH values were identified as a potentially significant selection regions (Fig. 5B).

### Landscape genomics analysis to identify environment-associated SNPs

The identification of markers linked to environmental variables was conducted using MatSAM v1.0. Software [19]. Rather than relying on theoretical models in population genetics, this spatial analysis employs spatial coincidence to establish a connection between the genetic makeup of the goats under study and the environmental parameters derived from the geographic coordinates of their sampling sites. In the analysis, a matrix is used where each row represents an individual and their geographic coordinates of sampling [4, 20]. To examine how allele frequencies relate to environmental parameters, a univariate logistic regression analysis is carried out at the individual genotype level [19]. The significance of the associations is determined using both the log-likelihood (G) test and a Wald test [20]. The Bonferroni correction is utilized to adjust for multiple comparisons [19]. By assessing the significance of models resulting from all potential pairwise combinations (allele vs. environmental parameter), statistically significant markers can be pinpointed. These specific loci are probable targets of selective sweeps driven by environmental adaptations [11].

Furthermore, an additional algorithm was utilized within the LFMM program to evaluate the relationships between SNPs and climate variables (http://membres-timc.imag.fr/Eric.Frichot/lfmm/index.htm) [21]. The LFMM method, a combination of population genomics, ecological modeling, and statistical learning, has been demonstrated to be successful in recognizing indications of local adaptation in genomes [4, 11, 26]. In addition, this method reduces the probability of false-positive associations caused by population structure and other random factors [40]. A principal component analysis (PCA) of environmental variables was conducted. The first principal component (PC1) explained the most variance (70.78%) compared to the second component (12.31%) (Fig. 3B). The first axis of the PCA was employed to summarize the variables. Subsequently, The Latent Factor Mixed Model (LFMM) algorithm was utilized to determine z-scores for all single nucleotide polymorphisms (SNPs) after 100 burn-in sweeps and 1,000 additional sweeps. Our approach involved incorporating K = 3 latent factors identified through population structure analyses carried out using the SmartPCA tool from the EIGENSOFT v5.0 package and the Bayesian clustering program STRUCTURE v2.3.4 [4, 11].

### Identification of candidate genes associated with selection signature

Following the assessment of the results, SNPs that exceeded the top 0.1% percentile threshold from all four methods were subjected to the intersection of multiple-selective signal analysis. Employing the BioMart tool [41] and the goat reference genome assembly (ARS1) [42], gene mapping in goats involved extracting 10 kb up- and downstream regions for each significant SNP in the overlapping regions. The protein-coding genes that overlap with regions experiencing positive selection have been pinpointed as potential candidate genes. The Database for Annotation, Visualization, and Integrated Discovery (DAVID) v6.8 was employed for gene enrichment analyses to facilitate further examination [43]. This database permits the examination of the Kyoto Encyclopedia of Genes and Genomes (KEGG) pathway and Gene Ontology (GO) for biological processes. The significance of enriched GO biological processes, molecular functions, and cellular components was determined using the Fischer test (*p*-value = 0.05). Furthermore, an exhaustive literature review was undertaken to elucidate the functions of the identified genes.

## Results

### Relationships between breeds based on climate variables and genomic data

To identify a cluster of distantly related breeds adapted to extreme environments, Principal Component Analysis (PCA) was conducted using climatic variables (Fig. 3A and B) and genetic relationships based on Reynold genetic distance between breeds (Fig. 3C). In this subset, it was anticipated that signs of climatic adaptation would be more pronounced and readily discernible, while signals of common origin among breeds would be diminished. PCA effectively grouped the 51 native goat breeds based on their adapted environments. PC1 and PC2, the first two principal components, collectively accounted for over 80% of the total variance, with PC1 explaining 70.78% and PC2 explaining 12.31% (Fig. 3B). PC1 distinguished between breeds based on the impacts of different environmental climate variables, while PC2 and PC3 did not show evident geographical divergence associated with these variables (Fig. 3A). The PCA analysis revealed that 24 breeds from Africa and West Asia clustered together in Cluster I due to their negative PC1 values, while 27 breeds from Europe, America, and Oceania formed Cluster II with positive PC1 values (Fig. 3A and B). Furthermore, through the use of a Neighbor-Net graph and Reynolds genetic distance, the 51 goat breeds were divided into two distinct clusters, in accordance with existing research on the phylogeography of these breeds [24]. The PCA plot using environmental variables indicated that breeds in Cluster I had negative PC1 values, while those in Cluster II had positive PC1 values (Fig. 3C).

### Detection of selection signatures

**Fig. 6:**
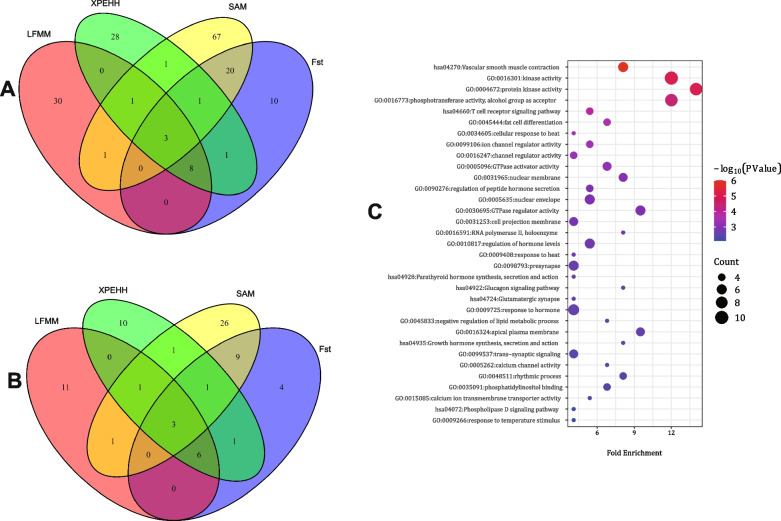
Venn diagram for SNPs and genes identified by Fst, XPEHH, LFMM, and SAM methods (**A** and **B** respectively) and GO and KEGG terms related to environmental adaptation (**C**)

To identify potential selection signatures across the genome, Fst and XPEHH tests were employed to analyze the distinctions between the goat populations in Cluster I and Cluster II. These tests have proven highly effective in detecting signatures with either nearly fixed or fully fixed alleles. The threshold for determining outliers was set at the top 0.1% SNP values, with the threshold values being 0.429028 for Fst and 0.278427 for XPEHH (Tables S2 and S3; Fig. 5A and B). The Fst test revealed 24 genes associated with various traits, including coat color, reproduction, feed intake, hair follicle development, heat adaptation, lipid metabolism, and neuronal development. Similarly, the XPEHH test identified 23 genes linked to feed intake, heat adaptation, fat deposition, milk traits, and coat color ((Table S6). Remarkably, 14 SNPs were concurrently detected by both the Fst and XPEHH tests (Fig. 6A). A more detailed examination of overlapping SNPs within a 5,000 bp range (SNP ± 5,000 bp) pinpointed 11 genes (*DENND1A*, *ITPR2*, *PLCB1*, *PREX2*, *ASIP*, *DLG1*, *GFI1*, *TBC1D12*, *TP53BP1*, *WDR75*, and *UVRAG*) of particular significance (Fig. 6B and Table S6).

Among these, *PLCB1* [44], *WDR75* [45], and *ITPR2* [13] are associated with heat tolerance, while *DLG1* [46] and *GFI1 *[47] play roles in feed intake and glucose homeostasis, respectively. Furthermore, *TP53BP1* [48], *ASIP* [48, 49], and *PREX2* [50] genes are linked to coat color and hair follicle development. *TBC1D12* is related to environmental stress [4, 51], *UVRAG* genes pertain to ultraviolet resistance [52], and *DENND1A* [14] is associated with reproduction (Table S6).

### Signatures of genomic adaptation to local environments

Two distinct approaches, LFMM and SAM, were employed for landscape genomics analyses (LGA) using climatic variables representing the current climate. With the LFMM method, 22 genes were successfully identified from the top 0.1% SNP values (Fig. 5C, Tables S4, and S6). Notably, among these 22 genes, 10 genes (*ASIP*, *DENND1A*, *DLG1*, *DNAJC16*, *GFI1*, *ITPR2*, *PLCB1*, *TBC1D12*, *TP53BP1*, and *WDR75*) were also detected by the Fst and XP-EHH tests. These genes are essential for coat color, feed intake, immune response, and heat tolerance, which makes them vital for local goat breeds to adapt to their respective climates. In the case of SAM, the 1000 highest WaldScore values were identified as potential SNPs, revealing 94 SNPs with significant associations to one or more climate variables (Fig. 6A and B, Tables S5 and S6). These 94 SNPs, considering a 5,000 bp range (SNP ± 5,000 bp), led us to the discovery of 42 genes. Importantly, three of these genes (*DENND1A*, *PLCB1*, and *ITPR2*) were also identified by LFMM, Fst, and XPEHH tests (Table S6).

### GO enrichments and KEGG analysis

Applying Fst, XPEHH, LFMM, and SAM strategies, 74 genes were identified, 32 of which were consistent and appeared in at least two of the techniques (Table S6). Subsequently, we used DAVID v6.8 to analyze these genes and discovered a variety of biological terms and pathways related to environmental adaptation (Fig. 6C and Table S6). Among the 148 GO and KEGG terms discovered, "vascular smooth muscle contraction" stood out as the most significant (Fig. 6C and Table S7). This term encompasses six genes (*BRAF*, *ITPR2*, *PRKG1*, *CALCRL*, *PLCB1*, and *ARHGEF12*). Furthermore, numerous essential terms were found related to environmental adaptation, such as "cellular response to heat" (including *TRPV1*, *TFEC*, *ANO1*), "GTPase regulator activity," "response to heat" (involving *CHN1*, *TBC1D12*, *PLCB1*, *ARHGEF12*, *DENND1A*, and *PREX2*), "rhythmic process" (with *PDGFRA*, *SREBF1*, *USP2*, *ENOX1*), and "response to temperature stimulus" (including *TRPV1*, *TFEC*, *ANO1*) (Fig. 6C and Table S7). Moreover, terms related to endocrine regulation and energy metabolic responses played a pivotal role in local adaptation. These included "fat cell differentiation" (*PDGFRA*, *SREBF1*, *PLCB1*, *NOC3L*), "regulation of peptide hormone secretion" (involving *SLC2A2*, *SREBF1*, *PLCB1*, *ANO1*), "regulation of hormone levels" (with *GFI1*, *PDGFRA*, *SLC2A2*, *SREBF1*, *PLCB1*, *ANO1*), "glucagon signaling pathway" (in which *ITPR2*, *SLC2A2*, and *PLCB1* participated), "response to hormone" (including *ASIP*, *MAPK14*, *RBBP5*, *SREBF1*, *TRPV1*, *PLCB1*, *MMS19*), and "negative regulation of lipid metabolic process" (with *ALK*, *GFI1*, and *SREBF1*) (Fig. 6C and Table S7). These findings reveal the diverse biological pathways and processes implicated in the adaptation of goat populations to their local environments, offering valuable insights into the genetic mechanisms underpinning these adaptations.

## Discussion

The study of goat environmental adaptation has historically been hampered by limited methodologies and data availability. Previous research often lacked comprehensive environmental information or employed narrow approaches, particularly noticeable in studies focusing on African goat breeds [6, 26]. In contrast, our study utilized four diverse strategies to identify genes related to adaptation in goats from Europe, Africa, Asia, America, and Oceania. Our genome scan has allowed us to analyze SNP variation in conjunction with environmental variables on a large scale, and provide a deeper insight into natural selection in response to climatic changes of goat breeds. In addition, the 51 goat breeds were divided into two clusters based on climatic factors. Cluster I encompasses primarily breeds from Africa and West Asia, where exhibit high temperatures, limited rainfall, and arid, hot conditions year-round. In contrast, Cluster II comprises mainly breeds from Europe, American, and Oceania, characterized by mild, rainy climates with warm winters, cool summers, and relatively high precipitation. Thus this integration of genetic and environmental data offers a more comprehensive understanding of the genetic mechanisms that potentially underlie the adaptation of goat breeds to their specific local environments.

Firstly, these studies found the importance of coat color and hair follicles for goat adaptations to climate conditions [8, 53]. Specifically, several important genes were identified, such as *HOXC13*, *PDGFRA*, *ASIP*, *PREX2*, and *LDLRAD*4 were associated with the development of coat color and hair follicles (Table S6). Notably, *HOXC13* showed a correlation with altitude and temperature variations in Tibetan cashmere goats [53]. Copy number variations (CNVs) at the *ASIP* locus have been established as causal variants influencing coat color phenotypes across diverse animal species, including cattle [49], sheep [54], and goats [55]. The genetic determination of coat color holds adaptive significance concerning climatic variations. Differential thermoregulatory responses are observed across colors, with breeds exhibiting dark coats showing enhanced absorbed heat compared to those with lighter or white coats, reflecting 50% to 60% of direct solar radiation [9]. The skin penetration of this absorbed heat is contingent upon coat structure and coloration [3]. In cold climates, goats tend to possess a higher density of hair follicles, contributing to a denser and warmer coat, thus providing insulation against low temperatures [3].

Secondly, this study has identified several significant genes associated with energy metabolism in goats (Fig. 6C). Notably, genes like *DLG1*, *ENOX1*, *GPC5*, *DNAJC16*, *FTO*, *GFI1*, and *SLC2A2* play essential roles in regulating feed intake and glucose metabolism. Additionally, genes like *PAFAH1B2*, *STK32B*, *MIR33B*, *SREBF1*, *GPCPD1*, and *ACSM1* are involved in lipid metabolism, while *WDR75*, *SCN7A*, and *PLCB1* contribute to thermo tolerance (Table S6). Our findings suggest that the acclimatization of indigenous goat breeds to harsh climates is predominantly influenced by intricate, interconnected energy metabolic reactions, similar to those documented in sheep [4, 26]. Climate has a profound impact on animal physiology and fitness, particularly among ruminants [56]. Climate variables such as sunlight, precipitation, and temperature have an indirect influence on the digestibility, quality, and quantity of forage, which subsequently has a significant impact on goats [57]. These climate variables can directly affect goats through thermoregulation [12], but the stronger effects are expected to operate indirectly by regulating plant quality and biomass [58].

In response to thermal stress, animals regulate their energy metabolism by adjusting feed intake in terms of variety and quantity when they deviate from the optimal body temperature range for cellular processes [59, 60]. Moreover, animal morphology displays variations, including body size, that align with essential thermoregulatory principles to manage body energy in different climates effectively. For example, Sudanese and Egyptian desert goats have relatively large to medium body size, which helps for evaporative heat loss [61]. Goats display better heat stress resilience than cows and sheep, showcasing adaptive feeding behaviors in warm climates. Specifically, Fawn goats demonstrate unique feeding habits compared to Saanen x hair goats, which suffer from increased heat stress and insufficient nutrient consumption [62]. Therefore, the energy metabolic adaptations of native goat breeds are strongly influenced by climate, encompassing both direct and indirect effects. These findings shed light on the intricate interplay between climate, energy metabolism, and breed-specific traits.

Moreover, our findings provide evidence for the selection of genes related to endocrine regulation (eg, response to hormone), rhythmic process (*PDGFRA*, *SREBF1*, *USP2*, and *ENOX1*), reproductive (*DENND1A*, *ALK*, *KIF1B*, and *KHDRBS2*) and nervous system (eg, presynapse) on goat physiology and evolutionary success (Fig. 6C and Table S7). The duration of daylight is crucial for goats to adapt to seasonal cycles. Sunlight duration is often responsible for the production of endocrine hormones, consequently affecting physiological activities, for example, the timing of reproduction in mammals [63]. The majority of goat breeds are seasonal breeders, and the onset of this reproductive cycle is triggered by the input of sunlight [63, 64]. This light serves as a cue to initiate a sequence of physiological processes, culminating in the secretion of a gonadotropin hormone. Thus, sunlight is a pivotal factor in the priming of the neuroendocrine axis of goats for reproduction [63]. Recent rapid climatic changes have significantly altered the seasonal events of goats, particularly their reproduction, leading to selective pressures on the perception of sunlight and its hormonal regulation [56].

In addition, our study demonstrates that climate can indirectly impact the regulation of autoimmune responses in various goat breeds through its influence on their habitats [65, 66]. Several novel genes (*LPP*, *DNAJC16*, *SUGT1*, *STARD10*, *STX2*, *CCR9*, and *BANK1*) have been identified as being associated with disease resistance and immune response for goat populations. For example, *LPP* is implicated in paget disease [67], *DNAJC16* plays a crucial role in coordinating immune responses [68], and *SUGT1* provides resilience against Haemonchus contortus [69]. Additionally, *STARD10* is linked to resilience against Paratuberculosis [70], *STX2* against Escherichia coli [71], *CCR9* shows potential for treating inflammatory bowel disease [72], and *BANK*1 regulates innate immune signaling in B cells [73].

The identification of identical candidate Single Nucleotide Polymorphisms (SNPs), genes, and genomic regions under selection through different methodologies can offer strong support for selective signatures [74]. Through the utilization of a strategy that merges complementary statistical methodologies, this study enables the identification of alleles undergoing subtle frequency shifts due to selection, while concurrently mitigating the occurrence of false-positive associations [21]. This strategic approach allows for the identification of new loci where SNPs display subtle yet consistent patterns across populations. While it is acknowledged that false positives can occur, especially when employing various statistical methods, we believe that the occurrence of such false positives would be minimal. Our approach provides a more refined and nuanced perspective on the selective signatures present in the genomic data, offering valuable insights into the genetic adaptations of the populations under study.

Our study is constrained by several limitations. The signals of natural selection that we observed are probably the result of both the direct and indirect influences of climate on the goat genome, given that several other environmental variables are partially associated with climatic factors. The complexity of overlapping environmental and ecological variables makes it difficult to distinguish the causal selective pressures from other influences. When analyzing the data, it is crucial to recognize that the environment may vary for each breed within the habitat region, and it is worth noting that our SNP data is limited to a small portion of the goat genome. Therefore, to gain a better understanding, more detailed environmental data and higher density SNPs are needed for each breed throughout the habitat region.

## Conclusions

In summary, our study explored the genetic basis of climate-induced adaptations in goat breeds through genome-wide scanning. This research pinpointed 74 candidate genes associated with local adaptation in goats, enriched in key Gene Ontology terms related to energy metabolism, endocrine regulation, rhythmic processes, and heat response. These findings enhance our knowledge of the genetic framework of climate-driven adaptive evolution. Moreover, they hold significant implications for the formulation of conservation strategies to tackle the impacts of swift global climate change on goat and other related livestock species.

### Supplementary Information

**Additional file 1: Table S1.** Goat sample information and climate data. **Table S2.** The top 1% candidate SNPs (red color) under selection identified by the Fst method. **Table S3.** The top 1% candidate SNPs (red color) under selection identified by XPEHH method. **Table S4.** The top 1% candidate SNPs (red color) under selection identified by the LFMM method. **Table S5.** Top 1000 SNPs associated with environmental variables identified by SAM method. **Table S6.** 74 candidate genes identified by four method (Fst, XPEHH, LFMM, and SAM). **Table S7.** 148 go and kegg terms identified by 74 candidate genes.

## Data Availability

The data and computing programs used in this manuscript are available from publicly published databases (https://datadryad.org/stash/dataset/doi:10.5061/dryad.v8g21pt).
